# Decoding Neuronal Ensembles in the Human Hippocampus

**DOI:** 10.1016/j.cub.2009.02.033

**Published:** 2009-04-14

**Authors:** Demis Hassabis, Carlton Chu, Geraint Rees, Nikolaus Weiskopf, Peter D. Molyneux, Eleanor A. Maguire

**Affiliations:** 1 Wellcome Trust Centre for Neuroimaging, Institute of Neurology, University College London, 12 Queen Square, London WC1N 3BG, UK; 2 Institute of Cognitive Neuroscience, University College London, 17 Queen Square, London WC1N 3AR, UK; 3 Lionhead Studios, 1 Occam Court, Surrey Research Park, Guildford, Surrey GU2 7YQ, UK

**Keywords:** SYSNEURO

## Abstract

**Background:**

The hippocampus underpins our ability to navigate, to form and recollect memories, and to imagine future experiences. How activity across millions of hippocampal neurons supports these functions is a fundamental question in neuroscience, wherein the size, sparseness, and organization of the hippocampal neural code are debated.

**Results:**

Here, by using multivariate pattern classification and high spatial resolution functional MRI, we decoded activity across the population of neurons in the human medial temporal lobe while participants navigated in a virtual reality environment. Remarkably, we could accurately predict the position of an individual within this environment solely from the pattern of activity in his hippocampus even when visual input and task were held constant. Moreover, we observed a dissociation between responses in the hippocampus and parahippocampal gyrus, suggesting that they play differing roles in navigation.

**Conclusions:**

These results show that highly abstracted representations of space are expressed in the human hippocampus. Furthermore, our findings have implications for understanding the hippocampal population code and suggest that, contrary to current consensus, neuronal ensembles representing place memories must be large and have an anisotropic structure.

## Introduction

Information about the environment is thought to be encoded in the brain by activity in large populations of neurons [1–3]. In order to understand the properties and dynamics of population codes, it is necessary to specify how they can be decoded in order to extract the precise information that they represent [2]. This enterprise is at the heart of neuroscience and provides a substantial challenge [3]. Decoding the activity of single, or small numbers of, neurons has been highly successful, with the best characterized example being the memory-related response of hippocampal place cells that fire invariantly when an animal is at a particular spatial location [4–6]. It is not clear, however, what information such place cells represent at the population level, given that recording in vivo from thousands of hippocampal neurons simultaneously is not currently possible [3, 7–9]. Other techniques such as immediate early gene imaging have provided some insights into memory representations at the population level [10, 11] but have limited temporal resolution (in the order of minutes) and do not provide an in vivo measure, making it difficult to isolate with precision the specific feature of a stimulus, memory, or behavior associated with gene expression.

Recently, invasive approaches to examining how neurons encode information [5, 12] have been complemented by multivariate pattern analyses of noninvasive human functional MRI (fMRI) data [13, 14]. Functional MRI measures signals that are indirectly correlated with neuronal activity simultaneously in many individual voxels. Each voxel, depending on its size and location, contains thousands of neurons. Conventional univariate fMRI analysis methods focus on activity in each individual voxel in isolation. In contrast, multivariate pattern analyses harvest information from local patterns of activity expressed across multiple voxels and, hence, large neuronal populations. Not only can such novel analyses infer the presence of neuronal representations previously thought below the spatial resolution of fMRI [15, 16], but the ensemble activity of such distributed patterns can predict the perceptual state or intention of an individual with high accuracy [17]. However, to date, there has been only limited application of this approach to memory [18] and none that has focused specifically on decoding activity in the hippocampus, despite its critical mnemonic role [19]. This is, perhaps, not surprising because making discriminations on the basis of activity in the hippocampus and surrounding medial temporal lobe (MTL) regions only presents a far more challenging classification problem than simply using whole-brain information in a category-based design that results in large activity differences across multiple brain regions [18].

However, successful decoding from focal hippocampal fMRI signals would have significant implications for understanding how information is represented within neuronal populations in the human hippocampus and for appreciating fundamental properties of the hippocampal population code. The current consensus from invasive animal studies [10, 11] and computational models [20, 21] is that this population code is random and uniformly distributed, casting doubt on some earlier studies that suggested a potential functional structure in the hippocampus [22, 23]. However, if there is a functional organization to the hippocampal population code, then activity at the voxel level should also be nonuniform, making classification possible with multivariate methods applied to human fMRI data [13, 14].

**Figure 1:**
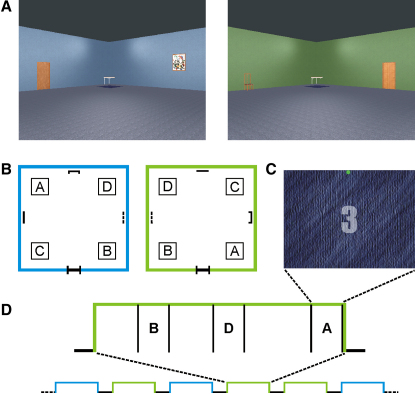
The Experimental Task (A) The virtual reality environment comprised two separate and distinct environments, a blue room and a green room. Each room was 15 m × 15 m and contained four “target” positions, which participants were instructed to navigate between as quickly and accurately as possible following extensive pretraining. (B) Schematic of the room layouts with the four target positions, labeled A, B, C, and D. These targets were visually delineated by identical cloth rugs (i.e., not by letters, which are depicted here only for ease of reference) placed on the floor at those positions and each 1.5 m × 1.5 m. Single objects (door, chair, picture, and clock with different exemplars per room but of similar size and color) were placed along the center of each wall to act as orientation cues. Identical small tables were placed in all four corners of the rooms to help visually delineate the wall boundaries. Single trials involved participants being instructed to navigate to a given target position with a keypad. The trial order was designed to ensure that the number of times that a target position was visited starting from another target position was matched across positions to control for goal and head direction. Once the intended destination was reached, the participant pressed a trigger button, causing the viewpoint to smoothly transition to look vertically downward at the floor (as if bowing one's head) to reveal the rug on the floor marking the target position, shown in (C). (C) At this point, a 5 s countdown was given, denoted by numerals displayed in white text overlaid on the rug (the number “3” is shown here as an example) and followed by the text label of the next target position (i.e., “A,” “B,” “C,” or “D”). The viewpoint then smoothly transitioned back to the horizontal, and navigation control was returned to the participant. (D) Environment blocks in each room consisted of two to four navigation trials and were counterbalanced across participants.

We set out to test this hypothesis by combining fMRI at high spatial resolution with multivariate pattern analysis techniques [13, 14, 24] to investigate whether it was possible to accurately predict the precise position of an individual within an environment from patterns of activity across hippocampal voxels alone. We used an interactive virtual reality (VR) spatial navigation task (Figure 1), given that spatial navigation critically relies on the hippocampus [4, 19]. Importantly, by holding visual inputs and task constant after successful navigation to a position within the VR environment, we could isolate and characterize the “abstract” (i.e., independent of current sensory inputs) internal representation of the environment's layout. With this approach, we show that noninvasive in vivo measurements of activity across the population of neurons in the human hippocampus can be used to precisely decode and accurately predict the position of an individual within their environment.

## Results

**Figure 2:**
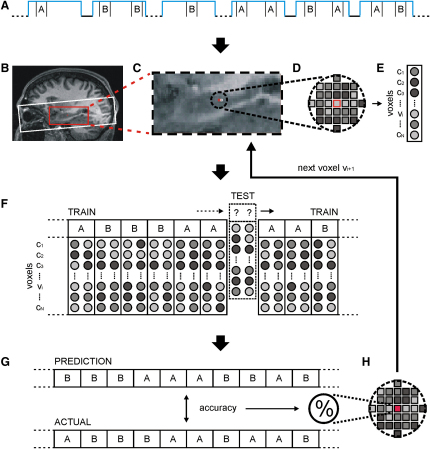
Multivariate Pattern Analysis An example multivariate analysis of a pairwise position classification, in this case discriminating between position A and position B in the blue room (see Figure 1). (A) Only volumes acquired while the participant was standing at these two blue room positions were entered into the analysis. (B) Coverage for functional scanning is shown as a white bounding box. The search space for the searchlight algorithm [14, 24], anatomically defined to encompass the entire hippocampus and wider MTL bilaterally, is shown as a red bounding box. (C–E) The search space was stepped through voxel by voxel (C). For each voxel v_i_ (example v_i_ outlined in red), a spherical clique (radius 3 voxels) of N voxels c_1.N_ was extracted with voxel v_i_ at its center (D) to produce an N-dimensional pattern vector for each volume (E). (F) Each pattern vector was labeled according to the corresponding experimental condition (position A versus position B) and then partitioned into a training set (solid lines) and an independent test set (dashed line and indented). Patterns of activity across the voxel clique from the training set were used to train a linear SVM classifier, which was then used to make predictions about the labels of the test set. A standard k-fold crossvalidation testing regime was implemented, ensuring that all pattern vectors were used once as the test data set. (G and H) This crossvalidation step, therefore, yielded a predicted label for every pattern vector in the analysis that was then compared to the real labels to produce an overall prediction accuracy for that voxel clique (G). This accuracy value was stored with the voxel v_i_ for later thresholding and reprojection back into structural image space (H). The whole procedure was then repeated for the next voxel v_i+1_ (outlined in white in [C]) along in the search space until all voxels in the search space had been considered.

We acquired blood-oxygen level-dependent (BOLD) contrast, high spatial resolution fMRI images focused on the hippocampus and wider MTL (see the Experimental Procedures and Figure 2B) while participants navigated as quickly and accurately as possible between four arbitrarily chosen target positions (A, B, C, and D) in each of two well-learned virtual reality environments: a blue room and a green room (Figure 1). These two environments were designed to be austere to minimize the impact of extraneous sensory inputs. Apart from color, which acted as a simple, unambiguous retrieval cue for each room and is processed in extrastriate cortex [25], the two environments were well matched, with no significant difference between navigation times or overall time spent in either room (see Table S1 available online for behavioral findings). Prior to our main multivariate pattern analysis, a conventional univariate analysis [26] performed with a general linear model confirmed that there was no significant difference in average brain activity between the two environments or any of the positions even at liberal thresholds, which was as expected given their almost identical macroscopic characteristics (see Supplemental Results).

### Discriminating between Two Positions

We first investigated whether we could accurately predict where a participant was located within a room solely from the pattern of fMRI BOLD responses across multiple voxels in the hippocampus and MTL. To do this, we initially made comparisons between arbitrarily selected pairs of positions (A versus B and C versus D) in both rooms. Importantly, after navigation, when participants reached a target position, the default horizontal viewpoint transitioned smoothly downward by 90° so that the entire visual display was occupied solely by an identical view of the floor (Figure 1C). Critically, only volumes capturing fMRI activity during this stationary phase (Figure 1D) at the target positions when the participant was viewing the floor were entered into the analysis. This is a key aspect of our study design because visual stimuli such as objects and boundaries are known to be processed by the MTL [12, 27–30]. By removing visual input as a confounding factor, we were thus able to isolate the internal representation of spatial location as the only difference between conditions. Moreover, the task design (see Supplemental Data) controlled for other potential confounding psychological factors during this period, as confirmed in the debriefing. The imaging data were then divided into independent training and test sets (see Figure 2), with the former used to train a linear support vector machine (SVM) classifier (see Experimental Procedures). The performance of this classifier was evaluated by running it on the independent test data and obtaining a percentage prediction accuracy value.

**Figure 3:**
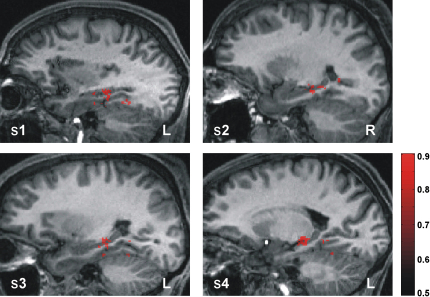
Pairwise Position Classification Prediction maps showing the accuracies of the voxels at the center of searchlight cliques that discriminate between two arbitrarily chosen target positions in a room (apriori selected to be A versus B and C versus D) significantly above chance (50%). The resultant prediction map for a participant, bounded by the search space (indicated by the red box in Figure 2B), is projected onto their structural brain image. A sagittal section for each participant is displayed, showing that voxels in the body-posterior of the hippocampus bilaterally are crucial for accurate position discrimination by the classifier. The findings are highly consistent across participants. The red bar indicates percentage accuracy values as a fraction (significance threshold set at 66.07% for all participants; see Tables S2 and S3 for thresholding and comparison pair details). “R” and “L” are right and left sides of the brain, respectively.

By using a multivariate “searchlight” approach to feature selection [14, 17, 24], we stepped through a large search space encompassing the MTL (Figure 2) and identified spherical cliques of voxels whose spatial patterns of activity enabled the classifier to correctly discriminate between two positions significantly above chance (p < 0.05 uncorrected, by using the statistically conservative approach of nonparametric permutation testing and accounting for the multiple comparisons problem [31, 32]; see Experimental Procedures and Tables S2 and S3). Voxels at the center of cliques whose accuracies survived this thresholding and were, therefore, important for accurately distinguishing between the two experimental conditions (e.g., position A versus position B) were then reprojected back onto the structural brain image of the participant to produce “prediction maps.” Remarkably, this process revealed large numbers of voxels in the body-posterior of the hippocampus bilaterally that accurately discriminated the position of the participant (Figure 3).

### Discriminating between Four Positions

**Figure 4:**
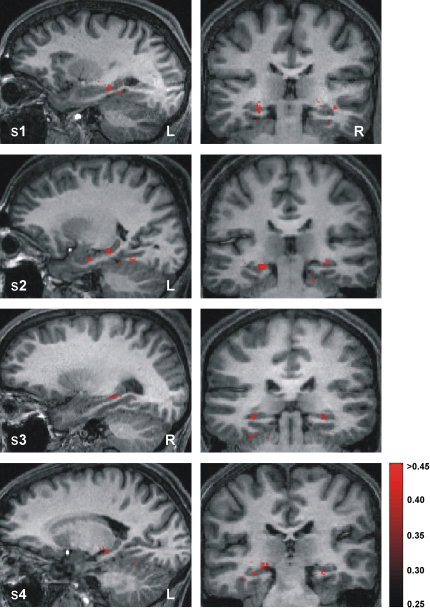
Four-Way Position Classification Prediction maps, bounded by the search space (indicated by the red box in Figure 2B) and projected onto each participant's structural brain image, showing the accuracies of the voxels at the center of searchlight cliques that discriminate between all four target positions in the same room significantly above chance (25%). Sagittal and coronal sections for each participant are displayed on left and right panels, respectively, showing that voxels in the body-posterior of the hippocampus bilaterally are crucial for accurate four-way position discrimination by the classifier. The findings are highly consistent across participants. The red bar indicates percentage accuracy values as a fraction (significance threshold set at 33.04% for all participants; see Tables S2 and S3 for thresholding details). Four-way position discrimination in the green room is shown for participants 1 and 2 and in the blue room for participants 3 and 4. “R” and “L” are right and left sides of the brain, respectively.

We next investigated whether there were voxels in the hippocampus capable of discriminating simultaneously between all four target positions in a room. By using the same protocol as above, we performed all six possible pairwise classifiers for each room (comparing positions A versus B, A versus C, A versus D, B versus C, B versus D, and C versus D against each other; see Figure 1) and combined their results into error correcting output codes from which resultant predictions were determined by computing the nearest Hamming distance to a real label code (see Supplemental Experimental Procedures). Although these four-way classifications are dependent on a linear combination of the pairwise classifications above, they provide distinct information about the data because significant voxel accuracy in pairwise classification does not necessitate significant accuracy in four-way classification. Significant voxels were again reprojected back onto the structural brain image of a participant to produce prediction maps. This revealed a focal cluster of voxels in the body-posterior of the hippocampus bilaterally, which allowed for accurate differentiation between all four positions in a room, again independent of visual input (Figure 4), a result that was markedly consistent across participants. There were very few discriminating voxels elsewhere in the MTL, thus demonstrating the specific involvement of the hippocampus in representing spatial positions.

### Discriminating between the Two Environments

**Figure 5:**
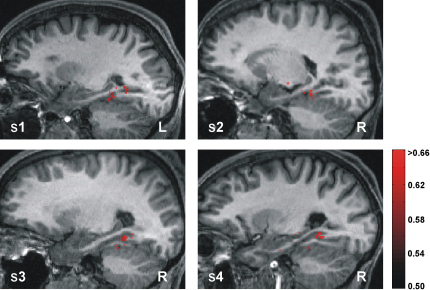
Environment Classification Prediction maps, bounded by the search space (indicated by the red box in Figure 2B) and projected onto each participant's structural brain image, showing the accuracies of the voxels at the center of searchlight cliques that discriminate between the blue room and the green room significantly above chance. A representative sagittal section for each participant is displayed, showing that voxels in the posterior parahippocampal gyrus bilaterally are crucial for accurate discrimination between the two environments by the classifier. The result is consistent across participants. Note the dissociation between the parahippocampal gyrus prediction maps here and the hippocampus prediction maps observed for position discrimination (see Figures 3 and 4). The red bar indicates percentage accuracy values as a fraction (significance thresholds were set for each participant between 57.45% and 58.00%; see Tables S2 and S3). “R” and “L” are right and left sides of the brain, respectively.

Though spatial positions of the participant within the environment were represented almost exclusively in the hippocampus, our findings also highlighted an interesting dissociation between the hippocampus and parahippocampal gyrus. In a separate multivariate analysis, we tested whether it was possible to accurately predict which environment—the blue or green room—a participant was in during navigation. The prediction maps obtained revealed voxels in the parahippocampal gyrus bilaterally, which allowed for differentiation between environments (Figure 5). In contrast to the position analysis, minimal numbers of voxels were found in the hippocampus that accurately discriminated between the two environments.

For each classification type, we formally quantified the differences in numbers of discriminating voxels present in the hippocampus and parahippocampal gyrus, respectively, by performing a difference of population proportions [33] significance test on the two anatomically defined regions (see the Experimental Procedures). For the pairwise and four-way position classifications, we found that there was a significantly higher proportion of voxels active in the hippocampus than the parahippocampal gyrus for all participants (all p < 0.05; see the Supplemental Results). For the environment classification, there was a significantly higher proportion of voxels active in the parahippocampal gyrus than the hippocampus for all participants (all p < 0.05; see the Supplemental Results). Note that these significant findings also mitigate against the multiple comparisons problem; if active voxels were just false positives due to chance, one would expect a uniform distribution of active voxels (see the Supplemental Results).

## Discussion

Our results demonstrate that fine-grained spatial information can be accurately decoded solely from the pattern of fMRI activity across spatially distributed voxels in the human hippocampus. This shows that the population of hippocampal neurons representing place must necessarily be large, robust, and nonuniform. Thus, our findings imply that, contrary to prevailing theories, there may be an underlying functional organization to the hippocampal neural code. Our data also revealed a dissociation, permitting conclusions about anatomical specificity. Whereas spatial positions were expressed in the hippocampus, by contrast, voxels in the parahippocampal gyrus discriminated between the two environments.

Extending the pairwise position classification findings (Figure 3) to discriminate between four arbitrary environmental positions (Figure 4) revealed a region of the hippocampus that is involved in the general storage and/or manipulation of position representations. The involvement of neuronal populations located specifically in the body-posterior of the hippocampus [19] as indicated by our data is highly consistent with findings from human and animal studies of spatial memory that use other investigative techniques [34–36]. Therefore, we propose that these individual abstracted position representations aggregated together form the basis of the allocentric cognitive map [4], or the set of invariant spatial relationships [37], representing the layout of an environment. Due to the constraint that pattern classifiers require a certain number of consistent examples for training purposes [13, 14], discrete localized positions had to be used as target locations. However, there is nothing special about the target locations used in this study; any positions in the rooms could have been chosen. Indeed, within each target location, a participant's stationary position varied subtly trial by trial, given that the target area measured 1.5 m × 1.5 m in size. Thus, we suggest that the spatial code for an environment is likely to be continuous, with subtle differences in the neuronal code between adjacent positions.

The volumes acquired during an environment block while in the blue or green room (see Figure 1D) comprised fMRI activity from a large number of different “snapshot” views of a room at numerous spatial positions within it (not only our four target positions). Hence, we believe that the classifier operating on hippocampal voxels did not discriminate between the two environments because this would have necessitated these voxels to have identifiably similar patterns of activity across environment block volumes (i.e., volumes acquired while in the blue or green room). However, hippocampal voxels were instead acutely tuned to individual spatial positions within a block and, therefore, displayed differing patterns of activity during navigation in an environment block that encompassed numerous spatial positions. By contrast, it is clear that the parahippocampal gyrus performed a distinct but complementary function. We speculate that this may have involved extracting the salient contextual features of each environment [27, 29], such as object-in-place associations [28] and orienting wall object configurations from multiple visual snapshots for input to the hippocampal place representations [30]. Thus, the classifier operating on parahippocampal gyrus voxels was able to discriminate between the two environments, although we cannot exclude the possibility that this region might have also been sensitive to the color differences between the two environments. Further studies will be needed to ascertain the exact nature and function of the representations in the parahippocampal gyrus during navigation and, indeed, in other neocortical areas such as the prefrontal and parietal cortices, which are also known to be involved in navigation [38] but were outside of the scanning coverage of this study.

The rigorous design of our paradigm—in particular, the careful matching of visual input at the destination locations, the counterbalancing of starting and destination location combinations, and the use of an incidental visual task to maintain attention during the stationary phase—allows us to conclude that any informative patterns of voxels found by our multivariate analyses must code for the internal representation of spatial location only and not for any other aspects of the task. In addition to these design features, our analysis was robust to any residual cognitive differences that may conceivably have occurred. Classifiers can be thought of as distinguishing between learned commonalities across multiple training examples of two experimental conditions. Therefore, in order for the classifier to successfully decode brain activity, the difference between two conditions must be systematic and consistent across the majority of the training examples. We carefully designed the paradigm to ensure that the only possible systematic difference between stationary periods was the internal representation of the current position. This was further confirmed by a number of additional control analyses that were performed to ensure that other factors such as the identity of the destination labels themselves or nearby orienting objects could not have significantly contributed to the successful decoding (see the Supplemental Experimental Procedures and Supplemental Results).

Hence, it is with some confidence that we can say that the hippocampal voxels that survived the rigorously controlled thresholding that we employed were associated with internal representations of position within the environment alone. A further point to note, specifically in relation to the effect of previously seen landmarks on the BOLD signal during the stationary phase, is that paths and approaches taken to target positions were not identical across trials and the timings of any views of landmarks en route varied widely. The effect of such substantial variability in paths to the target position in effect introduced a self-paced random jitter with respect to the influence of any landmarks seen on the BOLD signal during the stationary periods. Therefore, landmarks cannot be a contributing factor to the successful performance of the classifier on the position discrimination (see the Supplemental Results).

Our finding that it is possible to distinguish between well-matched spatial positions with human fMRI has significant implications for understanding the neuronal population code in the hippocampus. It has been proposed that information is encoded in the brain as a sequence of cell assemblies, with each activated clique encapsulating a fundamental unit of information [1–3]. Cell assembly synchronization is thought to take place over timescales of ∼30 ms [2], in contrast to the time frame of human neuroimaging, which measures activity averaged over ∼6 s. Although the BOLD signal is only an indirect measure of neuronal activity and there is ongoing debate about the relationship between the two [39], there is a robust correlation between BOLD responses and local field potentials [39, 40]. Therefore, patterns of voxel activations acquired during a single fMRI volume and capable of discriminating between well-matched positions are likely to reflect the average synaptic activity within many cell assemblies that, taken together, can represent high-level information such as spatial location within an environment.

Although neural codes in the hippocampus and wider MTL are generally considered to be “sparse” [12, 41], that term has been used to describe a wide range of different representational scales, from single “grandmother” cells [42] to more than two million cells in other accounts [41]. The human hippocampus contains ∼40 million principal neurons [19], and even at the high spatial resolution of the scanning employed here, this translates to ∼10^4^ neurons per voxel. Given the relatively coarse and noisy nature of human neuroimaging in both the temporal and spatial domains, it is striking that it was possible to robustly distinguish between positions of a participant in the environment that vary in only subtle ways. To the extent that multivariate classification with fMRI reflects biased sampling of a distributed anisotropic neuronal representation [16], our results are consistent with the notion that hippocampal neuronal ensembles representing place memories are large and have an anisotropic predictable structure. Moreover, the prediction maps that we obtained indicated the presence of information sufficient to decode position from voxels distributed spatially throughout the hippocampus. Our data, therefore, are broadly supportive of two previous invasive studies that have suggested that there may be some form of clustering [23] or topographical functional organization [22] in the hippocampus. Although numerous invasive studies have reported that the population code is random and uniformly distributed [10, 11], a point often implicitly assumed by computational models [20, 21], this would result in uniform patterns of activity at the voxel level, thus rendering classification impossible [13, 14]. However, there are ways in which these opposing views and our findings can be potentially reconciled. For instance, the spacing of tetrodes randomly sampling single neurons [11] could be out of phase with the structure of the underlying functional organization [22]. Disparate findings might also arise from differences in the clustering analyses used (see [23] compared with [11]). The effect of cell assembly synchronization on single-cell spike output may also be a contributing factor but is, as yet, largely unknown [2].

## Conclusions

Here, we focused on the cross-species behavior of navigation, demonstrating that highly abstracted representations of space are expressed across tens of thousands of coordinated neurons in the human hippocampus in a structured manner. In so doing, we have shown that, contrary to current consensus, neuronal ensembles representing place memories must be large, stable, and have an anisotropic structure. Spatial representations of the type investigated here have been suggested to form the scaffold upon which episodic memories are built [4, 30, 43], but the precise mechanism by which the hippocampus achieves this is still unknown. This crucial question is difficult to address in nonhumans, wherein even the existence of episodic memory has been challenged [44]. By showing that it is possible to detect and discriminate between memories of adjacent spatial positions, our combination of noninvasive in vivo high-resolution fMRI and multivariate analyses opens up a new avenue for exploring episodic memory at the population level. In the future, it may be feasible to decode individual episodic memory traces from the activity of neuronal ensembles in the human hippocampus. This brings ever closer the tantalizing prospect of discovering how a person's lifetime of experiences is coded by the neurons of the brain.

## Experimental Procedures

### Participants

Four healthy right-handed males with prior experience of playing first-person video games participated in the experiment (mean age 24.3 years, SD 3.2, age range 21–27). All had normal or corrected-to-normal vision. All participants gave informed written consent to participate in accordance with the local research ethics committee.

### Task and Stimuli

During scanning, participants were required to navigate as quickly as possible between four arbitrary target locations in two different virtual reality environments (Figure 1). The virtual reality environment was implemented with a modified version of the graphics engine used in the video game Fable (http://www.lionhead.com/fable/index.html). The room interiors were designed in the architectural package Sketch-up (http://sketchup.google.com) and imported into the graphics engine. The code for the environment, controls, and scanner pulse synchronization was written in C++ with Microsoft Visual Studio (http://msdn.microsoft.com/en-gb/vstudio/products/default.aspx). Participants controlled their movement through the environment with a four-button MRI-compatible control pad. The buttons were configured to move forward, rotate left, rotate right, and signal that a target destination had been reached. Participants were extensively trained in the VR environments prior to scanning (for details of the prescan training procedure, see the Supplemental Experimental Procedures). Each room was 15 m × 15 m, and perspective was set at the height of an average person, around 1.8 m above ground. The four target positions (A, B, C, and D) were situated 3 m in from the corners and visually delineated by identical cloth rugs. Each rug (and hence each target area) was 1.5 m × 1.5 m. Identical small square tables were placed in each corner to aid visibility and were irrelevant as cues for the navigation task. The two rooms were matched in terms of size, shape, luminosity, emotional salience, contents, and floor color. The rooms were designed so that spatial relationships between neighboring object categories as well as the target position labels were orthogonal for each room. Participants navigated through the rooms at a fast walking speed of 1.9 m/s. It was important for movement to be at a realistic speed and under participant control because self-motion is thought to play an important part in the spatial updating process [30, 45]. Hence, the use of interactive virtual reality was highly suited for extraction of position information that was as ecologically valid as possible.

Once a target location was reached, the viewpoint transitioned downward so that the identical floor texture occupied the entire field of view, thus ensuring that visual input was matched perfectly across positions. At this point, a 5 s countdown was given, followed by the letter of the next location, displayed for 2 s, during which time the participant was stationary and viewing the floor (“stationary phase”). The viewpoint then transitioned back to the horizontal, and the participant navigated to the next location as quickly and accurately as possible. Navigation blocks consisting of two to four individual trials were interspersed with a 13 s period of rest, during which a fixation cross was presented on a plain black screen. The label of the next target position was then displayed for 2 s before the participant was placed anew in one of the rooms with his back facing the closed door as if he had just entered the room. The trial and room orders were pseudorandomized and fully counterbalanced across participants. Each environment (i.e., blue or green room) was visited 20 times during the scanning session, giving 40 environment blocks in total. Within each room, every target position was visited 14 times, giving 112 trials in total. In order to maintain attention during the stationary countdown period, catch trials were included that involved an incidental visual task. The countdown numbers were displayed in white text, but occasionally one would flash red for 200 ms. Participants were instructed to press the trigger button as quickly as possible upon spotting a red number. There were eight catch trials spread throughout the scanning session—one at each target position and always at the end of a block. The volumes acquired during these catch trials were excluded from the analyses. After scanning, participants were debriefed and asked about the navigational strategies that they adopted (for details of the postscan debriefing procedure, see the Supplemental Data).

### Image Acquisition

A 3T Magnetom Allegra head scanner (Siemens Medical Solutions, Erlangen, Germany) operated with the standard transmit-receive head coil was used to acquire functional data with a T2^∗^-weighted single-shot echo-planar imaging (EPI) sequence (in-plane resolution = 1.5 × 1.5 mm^2^; matrix = 128 × 128; field of view = 192 × 192 mm^2^; 35 slices acquired in an interleaved order; slice thickness = 1.5 mm with no gap between slices; echo time TE = 30 ms; asymmetric echo shifted forward by 26 phase-encoding (PE) lines; echo spacing = 560 μs; repetition time TR = 3.57 s; flip angle α = 90°). All data were recorded in one single uninterrupted functional scanning session (total volumes acquired for each participant: s1 636 volumes; s2 640 volumes; s3 658 volumes; s4 670 volumes). An isotropic voxel size of 1.5 × 1.5 × 1.5 mm^3^ was chosen for an optimal tradeoff between BOLD sensitivity and spatial resolution. Further, the isotropic voxel dimension reduced resampling artifacts when applying motion correction. In order to minimize repetition time while also optimizing coverage of the regions of interest in the medial temporal lobe, we captured partial functional volumes angled at 5° in the anterior-posterior axis (see Figure 2B). Susceptibility induced loss of BOLD sensitivity in the medial temporal lobe was intrinsically reduced by the high spatial resolution and adjusting the EPI parameters for the given slice tilt (z-shim gradient prepulse moment = 0 mT/m × ms; positive PE polarity). A T1-weighted, high-resolution, whole-brain structural MRI scan was acquired for each participant after the main scanning session (1 mm isotropic resolution, 3D MDEFT).

### Imaging Data Preprocessing

This consisted of realignment to correct for motion effects and minimal spatial smoothing with a 3 mm FWHM Gaussian kernel. The first six “dummy” volumes were discarded to allow for T1 equilibration effects [26].

### Multivariate Pattern Classification

A standard univariate statistical analysis was performed with a general linear model implemented in SPM5 (www.fil.ion.ucl.ac.uk/spm) (for details of this analysis, see the Supplemental Experimental Procedures). We then performed a multivariate pattern analysis [13, 14] designed to identify brain regions where distributed fMRI activation patterns carried information about the environment that a participant was in or information about his position in that environment. A linear detrend was run on the preprocessed images to remove any noise due to scanner drift or other possible background sources [46]. Next, we convolved the image data with the canonical hemodynamic response function to increase the signal-to-noise ratio effectively acting as a low-pass filter [26]. BOLD signal has an inherent delay of around 6 s to peak response relative to stimulus onset due to the hemodynamic response function [26], and applying this convolution in effect delayed the peak by another 6 s, giving a total delay of 12 s. To best compensate for this delay, all onset times were shifted forward in time by three volumes, yielding the best approximation to the 12 s delay given a TR of 3.57 s and rounding to the nearest volume [13, 14]. The first volume and the last four volumes of each environmental block were discarded to allow for any orientation effects to settle (due to appearing suddenly in a room) and to exclude catch trials (always at the end of a block when present). Three separate multivariate classifications were carried out to (1) discriminate between which of two target positions in a single room the participant was standing (“pairwise”), (2) discriminate between all four target positions in a single room (“four-way”), and (3) discriminate between which of the two room environments the participant was in (“environment”). The same technique, described next, was used in all three types of classification.

In order to search in an unbiased fashion for informative voxels and maximize sensitivity, we used a novel variant of the “searchlight” approach [17, 24], a multivariate feature selection method [14, 24] that examines the information in the local spatial patterns surrounding each voxel v_i_ (Figure 2). This approach has another important advantage in that it results in statistical maps that allow for the anatomical mapping of the spatial pattern of informative voxels to be appreciated. Thus, for each v_i_, we investigated whether its local environment contained information that would allow accurate decoding of the current position. For a given voxel v_i_, we first defined a small spherical clique of N voxels c_1.N_ with a radius of three voxels centered on v_i_. A radius of three voxels was reported to be the optimal size for a clique by Kriegeskorte et al. [24], although this may be partially dependent on the resolution of the acquired images. For each voxel c_1.N_ in the fixed local clique, we extracted the voxel intensity from each image, yielding an N-dimensional pattern vector for each image. Multivariate pattern recognition was then used to assess how much position and environment information was encoded in these local pattern vectors. This was achieved by splitting the image data (now in the form of pattern vectors) into two segments: a “training” set used to train a linear support vector pattern classifier (with fixed regularization hyperparameter C = 1) to identify response patterns related to the two conditions being discriminated and a “test” set used to independently test the classification performance. The classification was performed with a support vector machine (SVM) [47] by using the LIBSVM implementation (http://www.csie.ntu.edu.tw/∼cjlin/libsvm/). We used a standard k fold crossvalidation testing regime [13, 15, 47], wherein k equaled the number of blocks, with each block set aside, in turn, as the test data and the rest of the blocks used to train the classifier (see Figure 2F). This procedure was then repeated until all blocks had been assigned once as the test data (the crossvalidation step). Thus, the pairwise position classification involved a 28-fold crossvalidation step (14 position X and 14 position Y miniblocks of two volumes each; because the stationary phase lasted 7 s and the scanning repetition time was 3.57 s, this consisted of the two volumes immediately following the onset of the stationary period); the four-way position classification involved a 56-fold crossvalidation step (14 miniblocks of length two volumes for each of four positions); and the environment classification involved a 40-fold crossvalidation step (20 blue room and 20 green room blocks, with an average of seven volumes per block). Every volume within a test block was individually classified following the crossvalidation step, thus yielding an overall percentage accuracy for the clique centered around voxel v_i_ for all of the volumes in the entire experimental session (see Figure 2G). This decoding accuracy was stored with voxel v_i_ for subsequent reprojection as a “prediction map” (see “Reprojection and Thresholding” below), and the entire procedure was repeated on a voxel-by-voxel basis until all voxels in a previously defined region of interest had been considered. In this case, the search regions were anatomically defined with two large rectangular bounding boxes (each composed of 6750 voxels; see Figure 2B) covering both the right and left medial temporal lobes and thus encompassing our apriori regions of interest, i.e., the hippocampus and parahippocampal gyrus. Good overall classification accuracy for a voxel v_i_ implies that patterns in the surrounding local clique of voxels encode information about the current position and environment of the participant. A final multiclass classification procedure was performed for the four-way position classification (see the Supplemental Experimental Procedures for details).

### Reprojection and Thresholding

Once the classifications were completed and the decoding accuracies were stored for each voxel in the search region, we proceeded to reproject these values back into structural brain image space to allow the resultant prediction maps to be visually inspected. These prediction maps were then thresholded at a percentage accuracy value that was significantly above that expected by chance. This significance threshold was determined by using the classical method of nonparametric permutation testing [31, 32], requiring minimal assumptions (for example, about the shape of the population distribution) for validity. The entire classification procedure outlined above was performed 100 times with a different random permutation of the training labels for each classification type for each participant. The individual voxel accuracy values from each of these 100 random runs were then concatenated into one population, and the accuracy value at the 95th percentile of this aggregated distribution was calculated. Therefore, this procedure yielded a percentage accuracy value for each individual participant, above which a voxel's accuracy was considered significant, equating to a confidence level of p < 0.05 uncorrected in a standard t test.

We accounted for the multiple comparisons problem by performing a standard test for the difference between two population proportions [33]. If significant voxels are false positives due to random variation, the proportion of significant voxels should be uniform over the entire search space (see Figure 2). To test this null hypothesis, we created two anatomical masks, one covering the hippocampus bilaterally and the other the parahippocampal gyrus bilaterally, for each individual participant by hand with MRIcro (http://www.sph.sc.edu/comd/rorden/mricro.html) by using each participant's structural MRI scan for guidance. The proportion of significant voxels for each region was determined (i.e., active voxels/total voxels) for each prediction map (i.e., pairwise position, four-way position in blue room, four-way position in green room, and environment) for each participant. A two-tailed test for difference between proportions was performed for each of the prediction maps to determine whether the proportion of active voxels in the hippocampus was significantly different from that in the parahippocampal gyrus.

In addition to the voxel count difference of proportions test described above, we used a second analytic approach to test for a region x classification type interaction between the hippocampus and parahippocampal gyrus. We assessed the informational content of BOLD signals for each type of classification (i.e., pairwise position and context) for both regions. A classifier trained on A versus B in the blue room (comprising primarily hippocampal voxels; see Figure 3) was tested on discriminating between the blue room and green room contexts. Conversely, a classifier trained on blue versus green room context (comprising primarily parahippocampal voxels; see Figure 5) was tested on discriminating between positions A versus B in the blue room. To enable comparison, we generated a single accuracy value for each classifier (see the Supplemental Experimental Procedures and Supplemental Results).

## Supplemental Data

Document S1. Supplemental Experimental Procedures, Supplemental Results, Five Tables, and One Figure
