# Supplementary material for: Decoding Neuronal Ensembles in the Human Hippocampus
Source: Curr Biol. 2009 Apr 14;19(7-3):546–54. doi: 10.1016/j.cub.2009.02.033 (PMC2670980; doi:10.1016/j.cub.2009.02.033)
Supplement: Document S1. Supplemental Experimental Procedures, Supplemental Results, Five Tables, and One Figure [file mmc1.pdf]

Current Biology, ***Volume 19***

Supplemental Data

Decoding Neuronal Ensembles in the Human Hippocampus

Demis Hassabis, Carlton Chu, Geraint Rees, Nikolaus Weiskopf,
Peter D. Molyneux, and Eleanor A. Maguire

SUPPLEMENTAL DATA

SUPPLEMENTAL EXPERIMENTAL PROCEDURES

Participants

We adopted a case-plus-replication approach standard in the literature (e.g. [1]), in which each of our four participants was treated as an independent case for the purposes of analysis, with detailed single case analyses followed by replication of the findings in each independent test case.

Prescan Training

Prior to scanning participants were pre-exposed to the two environments during an extensive training session where they were instructed to familiarize themselves with the keypad controls and were introduced to the navigation task. They were given no prior knowledge of the mapping of labels to target positions but were encouraged to learn through trial-and-error at their own pace. Feedback was given in the form of a red dot displayed centrally at the top of the screen when the trigger button was pressed over an incorrect target position. No suggestion was given to the participants to use any particular strategy for learning the two room layouts and they were instead encouraged to utilise whatever method worked for them. The training program periodically switched between the blue and green rooms to allow both layouts to be learned (training order

was counter-balanced across participants). Before proceeding to the scanner, participants had to reach criterion performance in a behavioural test where they had to correctly complete 10 navigation trials in a row in each room to ensure the two layouts had been well-learned. It took approximately 30 minutes of training for participants to reach this level of performance. The training protocol was carried out in order to minimise any learning or novelty effects during the scanning session and to allow time for the neural representations for each environment to form and stabilise [2].

Debriefing

After scanning, participants were debriefed. They were asked about the strategies they used to learn the room layouts initially and to navigate between target positions during scanning. They were then asked to rate their emotional response to each room on a scale from -3 (really dislike) to +3 (really like) and also how difficult they found the task on a scale from 1 (easy) to 5 (hard). Finally, the participants were asked about any other observations they had pertaining to the layouts, the room representations, and their overall concentration during the task.

Univariate Data Analysis

After imaging data pre-processing (see Experimental Procedures) a standard univariate statistical analysis was performed with a general linear model implemented in SPM5 (www.fil.ion.ucl.ac.uk/spm). The paradigm consisted of 20 blocks of trials in each of the blue and the green rooms, pseudorandomly ordered, lasting an average of 42.5s (SD 1.36s) resulting in the acquisition of approximately 12 volumes per block, and separated by rest periods of 15s (13s rest plus 2s next target indication – see above). Within an environment block the stationary period of each navigation trial was also separately modelled as a ‘mini-block’ consisting of the two volumes immediately following the start of the stationary period at the target position. There were 14 navigation trials for each of the four positions in the two rooms yielding a total of 112 position trials in total. Each

environment block and position mini-block was modelled as a boxcar function and convolved with the canonical hemodynamic response function to create regressors of interest and participant-specific movement parameters were included as regressors of no interest. Participant-specific parameter estimates pertaining to each regressor (betas) were then calculated for each voxel. Given our apriori interest in the MTL, a significance threshold of $p < 0.001$, uncorrected for multiple comparisons, was employed with an extent threshold of five or more contiguous voxels [3]. The data were also examined using a much more liberal threshold of $p < 0.05$ uncorrected for multiple comparisons.

Multiclass Classification

Standard SVMs are binary classifiers that operate on two-class problems. However, they can be arbitrarily extended to work in cases where there are more than two classes [4] as with the 4-way position classification performed here. Typically, this is done by reducing the single multiclass problem into multiple binary classification problems that can be solved separately and then recombined to provide the final class prediction [4, 5]. For the 4-way position classification problem in the present study we used the well-established approach of Error Correcting Output Codes [5, 6] to assign a unique binary string of length n to each of the 4 classes (class "codewords" representing positions A, B, C, D respectively) where n corresponds to the number of binary classifiers performed. There are 6 possible pairwise comparisons that can be made between 4 positions, therefore we performed all 6 pairwise binary classifications and combined their outputs to produce codewords 6-bits in length (see Supplemental Table 4 for the codeword matrix) with each bit representing the output from a single binary classifier. These output strings were then compared against all 4 of the pre-assigned class codewords to determine the final predicted class. This was achieved by computing the Hamming distance (i.e. the number of bits which differ between two binary strings) [7] between the output string and the class codewords to find the closest class codeword and thus by

association the predicted class. Apart from this extra multiclass procedure, the 4-way position classification was performed with the same methods as the other binary classifications (as described in the Experimental Procedures).

Additional Multivariate Control Analyses

In order for a classifier to successfully decode brain activity, the difference between two conditions must be systematic and consistent across the majority of the training examples. For example this is not the case for the navigation periods between locations which were self-paced and thus exhibited considerable trial-by-trial variability in terms of the paths taken and the objects viewed en route and thus cannot have been a contributing factor to successful classification at the destination locations. In order to empirically confirm this hypothesis, we performed a further binary classification analysis this time only using the volumes acquired during the navigation periods prior to reaching a destination location. We carried out this analysis for all four participants in the blue room on the navigation periods prior to reaching position A versus the navigation periods prior to reaching position B.

Whilst we believe our task is optimally designed to allow us to conclude that discrimination between within-environment locations reflects neural signals related to space, it is necessary to consider alternative, possibly confounding, factors. We performed a series of new analyses, following the same procedure in each case. We took the subset of hippocampal voxels that could discriminate between within-environment locations (i.e. those from the pairwise position classification) and now tested whether signals from these voxels could instead discriminate the alternative factors. If they could, then our conclusion that signals from these voxels encoded within-environment locations would be called into question.

Three new classification analyses were undertaken: (1) Destination labels: If a classifier learns to discriminate patterns of activity that represent the verbal label "A" in environment 1, does this classifier perform above chance in discriminating label "A" in environment 2? (2) Nearby objects: looking at Figure 1, the landmarks near locations B and A in the blue room resemble those of locations B and C in the green room. If a classifier learns to distinguish locations B and A in the blue room, does this classifier perform above chance in discriminating locations B and C in the green room? (3) Position relative to global 'room-centred' coordinates: if a classifier learns locations in the blue room can it discriminate corresponding locations in the green room? In this analysis 'locations' refer to spatial, room-centred coordinates, i.e. relative to the entry point (the door, which was the most-used orienting object). For each of the three analyses, we took the subset of voxels from our original analyses (see Figure 3) that had accurately discriminated within-room position, and now trained a new classifier on all the examples of the particular alternative factor selected for analysis in one of the rooms (e.g. A v B in the blue room). We then tested this new classifier on the discrimination between the corresponding confounding factor in the other room (e.g. A v B in the green room). Critically, this ensures that training and test data are independent and dissociates testing of these confounding factors (which are common to each room) from neural signals related to space. To enable comparison, we generated a single accuracy value for each participant and confounding factor (and for the original within-environment position discrimination using the same methodology).

For the destination label factor, we tested a classifier trained on A v B in the blue room on discriminating A v B in the green room. For the nearby objects factor, we tested a classifier trained on A v B in the blue room on discriminating B v C in the green room, as those pairs of positions had the same neighbouring object exemplars in both rooms.

Finally, for the relative position factor using global room-centred coordinates as anchored by the door, we tested a classifier trained on C v D in the green room on discriminating A v D in the blue room, as those pairs of positions had the same relative position in each room as viewed from the door. For the two participants who had counter-balanced trial positions in the blue and the green rooms, these were swapped for the purposes of training and testing the new classifiers.

SUPPLEMENTAL RESULTS

Behavioural Data

The blue and green rooms were well-matched in terms of design and layout (see Figure 1). Within each room target positions and trial types were also well-matched. This was confirmed behaviourally with no significant difference between time spent in each of the rooms ($p = 0.38$), or average times to navigate to each target position ($p > 0.48$ for all pairwise comparisons; see Supplemental Table 1).

Debriefing

Participants were encouraged to explain the strategies they used for learning the room layouts initially and how they performed the navigation task during scanning. All reported forming a 'mental map' of the each room's layout and only occasionally using one of the wall objects (three used the door, one used the clock) as an anchor point to maintain their bearings. All reported viewing their mental map from an overhead aerial viewpoint but two also reported being aware of the angle of the next target position from a first-person perspective during the navigation task. None reported using a verbal strategy or mentally verbalising the destination location labels during the task. The task was generally found to be quite easy [ratings from 1(easy) to 5 (hard), mean rating 2.0 (0.82)], and both rooms were equivalent in emotional salience with slightly positive feelings reported for both [-3 (really dislike) to +3 (really like); green room +0.75 (1.50), blue room +0.25 (0.96); p=0.59)]. All participants noticed the different object configurations in each room, treated each room as a separate environment (not spatially connected in any way), and were able to maintain concentration on the navigation task throughout.

Univariate Neuroimaging Data

First we characterised any activity differences associated with the two environments by contrasting the blocks of trials in the blue room with those in the green room. As expected, no significant differences in activity were detected anywhere in the brain for any of the participants. Next we investigated whether there were any activity differences between pairs of target position mini-blocks. Once again, no significant differences in activity were detected anywhere in the brain for any of the participants for any of the pairwise comparisons. This was the case even at an extremely liberal statistical threshold of $p < 0.05$ uncorrected for multiple comparisons. These null univariate results were expected because conventional univariate analysis works by measuring the difference in average voxel activity between conditions [3]. With conditions almost identically matched in terms of stimuli and task, it is no surprise that this method did not reveal any significant differences in average univariate activity, hence the advantage of using a multivariate approach sensitive to patterns of information across groups of voxels [8] (see Figures 3, 4 and 5).

Permutation Testing and Thresholds

Our multivariate searchlight analysis approach provided prediction accuracy values for every voxel in the search space (see Experimental Procedures). In order to determine in an unbiased fashion whether these accuracies were significantly above chance (50% in the case of environment and pairwise position classification and 25% in the case of the 4-way position classification) 100 independent runs of the classification were performed with random permutations of the training labels. Voxel accuracies from these runs were concatenated into one distribution and the 95th percentile found. This accuracy value then represented an unbiased threshold above which voxel accuracy was considered to be significantly above chance. Supplemental Table 2 summarises the threshold levels for each participant for each classification type. The overall consistency of the value of

the 95th percentile accuracy for the permutation testing across participants shows that 100 independent random runs were sufficient to account for any random statistical variation in the voxel populations whilst remaining computationally feasible on standard multi-processor computers [9]. Note that the environment thresholds show slight variability between participants compared to the position thresholds due to there being many more volumes to classify (~280 for environment compared to 56 for pairwise position and 112 for 4-way position) therefore allowing for a finer granularity of percentage accuracy values. The greater number of volumes to be classified is also the reason why the significance threshold for environment is lower than that for pairwise position.

We performed three final verification procedures. First, for every voxel in the search space we also averaged their individual accuracy values across all 100 random runs and found the 95th percentile of that distribution. Supplemental Table 3 summarises these threshold values and, as can be observed, they are at chance with no voxel accuracies reaching significance thus indicating there was no systematic noise bias in the underlying imaging data. Second, for each voxel individually we took the 95th percentile accuracy value across the 100 random runs for the 4-way classification in the blue room and plotted them (Supplemental Figure 1). For all participants the values were normally distributed round a mean of approximately 33% with a small standard deviation of 1.46% thus demonstrating that there is no systematic anatomical variation in the signal-to-noise ratio across the search space and justifying the use of a single global threshold value for all voxels in the search space.

To account for the multiple comparisons problem a test for the difference between two population proportions [10] was performed on two different anatomical regions of the

search space covering the hippocampus and parahippocampus respectively. For the pairwise (Figure 3) and 4-way (Figure 4) position prediction maps for both the blue and green rooms the proportion of active voxels in the hippocampus was significantly higher than for the parahippocampal gyrus in all participants (s1: pairwise, $z=2.707$, $p=0.007$; 4-way blue, $z=4.743$, $p<0.001$; 4-way green, $z=4.429$, $p<0.001$; s2: pairwise, $z=3.173$, $p=0.002$; 4-way blue, $z=2.317$, $p=0.02$; 4-way green, $z=10.201$, $p<0.001$; s3: pairwise, $z=11.361$, $p<0.001$; 4-way blue, $z=4.734$, $p<0.001$; 4-way green, $z=10.429$, $p<0.001$; s4: pairwise, $z=2.031$, $p=0.042$; 4-way blue, $z=2.694$, $p=0.007$; 4-way green, $z=3.157$, $p=0.002$). Conversely for the environment discrimination (Figure 5) the proportion of active voxels in the parahippocampal gyrus was significantly higher than for the hippocampus in all participants (s1: $z=-4.292$, $p<0.001$; s2: $z=-2.261$, $p=0.024$; s3: $z=-7.570$, $p<0.001$; s4: $z=-2.409$, $p=0.016$). These findings therefore quantify our conclusions on the dissociation observed in the activity patterns of the hippocampus and parahippocampal gyrus (see main text, Figures 3, 4 and 5) and also allow us to reject the null hypothesis and conclude that there is a significant difference in the proportions of active voxels between the hippocampus and parahippocampal gyrus. Thus our data cannot be explained by false positives due to random variation, as this would have resulted in a uniform distribution of significant voxels across the whole search space.

In addition to the voxel count difference of proportions test described above, we used a second analytic approach to test for a region x classification type interaction between the hippocampus and parahippocampal gyrus (see Experimental Procedures). Classifiers trained on A v B in the blue room (with average classification accuracy 66.1%, SD 6.4%, significantly above chance $p=0.015$) performed at chance when discriminating the blue room and the green room contexts (average classification accuracy 51.9%, SD 3.0%, not significantly above chance $p=0.27$). Similarly, classifiers trained on the blue room versus

green room context (with average classification accuracy 54.9%, SD 0.8%, significantly above chance p=0.001) performed at chance when discriminating A v B in the blue room (average classification accuracy 51.3%, SD 5.5%; not significantly above chance p=0.66). This therefore confirms that there is a region x classification type interaction between the hippocampus and parahippocampal gyrus and the position and context classifications.

Additional Multivariate Control Analyses

An additional control binary classification for all participants was performed on volumes acquired during the navigation period in the blue room prior to the reaching position A versus those acquired prior to reaching position B. The thresholded reprojections did not reveal any consistent patterns of results anywhere in the search space. Therefore it is very unlikely that the activation patterns observed on the basis of the volumes acquired during the stationary phase at the destination locations (see Figure 3 and Figure 4) could be due to BOLD signal generated by any orienting objects systematically viewed en route.

A further set of additional analyses were performed to confirm no other factors could have significantly contributed to the decoding performance (see Supplemental Table 5 for individual participant data). Classifiers trained on destination labels (e.g. A v B) in the blue room performed at chance when discriminating destination labels (e.g. A v B) in the green room (average classification accuracy 51.3%, SD 5.9%, not significantly above chance p=0.68) thus demonstrating that BOLD signals we measured did not encode destination labels as a discriminable factor in this task. Classifiers trained on nearby landmarks (e.g. A v B) in the blue room also performed at chance when discriminating corresponding nearby landmarks (B v C) in the green room (average classification

accuracy 52.5%, SD 5.6%, not significantly above chance p=0.45) thus showing that the similarity of nearby orienting object exemplars was not a discriminable factor from BOLD signals recorded in this task. Finally, classifiers trained on relative position (e.g. C v D in the green room) performed at chance when discriminating A v D in the blue room (average classification accuracy 47.3%, SD 8.6%, not significantly above chance p=0.58) thus demonstrating that the internal representation of spatial position was absolute and not relative to global room-centred coordinates anchored by the door.

References

1. Friston, K.J., Holmes, A. P., and Worsley, K.J. (1999). How many subjects constitute a study? Neuroimage *10*, 1-5.
2. Wilson, M.A., and McNaughton, B.L. (1993). Dynamics of the hippocampal ensemble code for space. Science *261*, 1055-1058.
3. Frackowiak, R.S.J., Friston, K.J., Frith, C.D., Dolan, R.J., Price, C.J., Zeki, S., Ashburner, J.T., and Penny, W.D. (2004). Human Brain Function (New York: Elsevier Academic Press).
4. Allwein, E., Schapire, R., and Singer, Y. (2001). Reducing multiclass to binary: a unifying approach for margin classifiers. Journal of Machine Learning Research *1*, 113-141.
5. Mourao-Miranda, J., Reynaud, E., McGlone, F., Calvert, G., and Brammer, M. (2006). The impact of temporal compression and space selection on SVM analysis of single-subject and multi-subject fMRI data. Neuroimage *33*, 1055-1065.
6. Dietterich, T.D., and Bakiri, G. (1995). Solving multiclass learning problems via error correcting output codes. Journal of Artificial Intelligence Research *2*, 263–286.
7. Hamming, R.W. (1950). Error detecting and error correcting codes. The Bell System Technical Journal *29*, 147-160.
8. Haynes, J.D., and Rees, G. (2006). Decoding mental states from brain activity in humans. Nat Rev Neurosci *7*, 523-534.

9 Nichols, T.E., and Holmes, A.P. (2002). Nonparametric permutation tests for functional neuroimaging: a primer with examples. Hum Brain Mapp *15*, 1-25.

10. Daniel, W.W., and Terrell, J.C. (1995). Business Statistics for Management and Economics (Boston: Houghton Mifflin).

Table S1. Behavioural Measures

Condition	Time (s) for each participant: mean (SD)				
	**s1**	**s2**	**s3**	**s4**	**Avg**
Blue Room					
Overall*	20.36(13.20)	20.65(13.47)	23.08(14.42)	23.73(13.86)	21.96(1.70)
Navigate to A	6.37(1.93)	5.92(1.05)	6.71(2.32)	7.02(1.83)	6.51(0.47)
Navigate to B	5.43(1.23)	5.95(1.01)	6.97(4.36)	6.97(2.89)	6.33(0.77)
Navigate to C	6.30(1.49)	6.08(1.68)	5.83(1.19)	7.12(1.98)	6.33(0.56)
Navigate to D	5.48(1.00)	6.22(1.19)	8.05(5.83)	7.45(3.95)	6.80(1.16)
Green Room					
Overall*	20.59(13.31)	20.19(13.23)	21.01(14.23)	21.34(12.97)	21.03(0.93)
Navigate to A	6.15(2.49)	5.92(0.83)	6.25(1.72)	6.77(1.32)	6.27(0.36)
Navigate to B	5.71(0.96)	5.79(0.96)	6.73(2.89)	6.18(1.05)	6.10(0.47)
Navigate to C	5.34(1.63)	6.07(1.07)	5.55(1.21)	7.24(1.47)	6.05(0.85)
Navigate to D	6.95(2.87)	5.75(0.76)	6.13(1.08)	6.42(2.52)	6.31(0.51)

*Overall times exclude the stationary phases

Table S2. 95th Percentile Accuracy Value for Prediction Map Thresholding*

Classification	95th percentile accuracy value (%) for each participant			
	s1	s2	s3	s4
Pairwise position†	66.07	66.07	66.07	66.07
4-way position (blue room)	33.04	33.04	33.04	33.04
4-way position (green room)	33.04	33.04	33.04	33.04
Environment	57.91	58.00	57.45	57.74

*Chance is 50% in the case of pairwise position and environment classification, and 25% in the case of the 4-way position classifications. The values in the table represent accuracy thresholds above which a voxel was considered significant, equivalent to a confidence level of $p < 0.05$ uncorrected in a standard t-test.

†For pairwise position classification target pairs were selected at random. Thus the 100 random runs were performed on: blue room AvB for s1; blue room AvB for s2; green room AvB for s3; blue room CvD for s4.

Table S3. 95th Percentile Averaged Accuracy Values for Random Runs*

Classification	95th percentile averaged accuracy value (%) for each participant			
	s1	s2	s3	s4
Pairwise position†	50.16	50.06	49.78	50.78
4-way position (blue room)	25.59	25.96	25.76	25.37
4-way position (green room)	25.32	25.58	25.66	25.53
Environment	49.45	50.03	49.51	49.92

*Chance is 50% in the case of pairwise position and environment classification, and 25% in the case of the 4-way position classifications. Note that all the averaged accuracy values in the table are at chance with no voxel accuracies reaching significance thus indicating there was no systematic noise bias in the underlying imaging data.

†For pairwise position classification target pairs were selected at random. Thus the 100 random runs were performed on: blue room AvB for s1; blue room AvB for s2; green room AvB for s3; blue room CvD for s4.

Table S4. 6-Bit 'Class Codewords' for 4-Way Position Classification

Position	Binary Classifier Output					
	(A v B)	(A v C)	(A v D)	(B v C)	(B v D)	(C v D)
A	1	1	1	0	0	0
B	-1	0	0	1	1	0
C	0	-1	0	-1	0	1
D	0	0	-1	0	-1	-1

'1' or '-1' indicates output from that binary classifier is expected to be class 1 (i.e. class listed first) or class 2 respectively. '0' indicates output from that binary classifier is considered neutral for that label assignment.

Table 5. Control Analyses

Participant	Classification accuracies			
	*Original (spatial)**		*Destination labels*	
S1	bAvB	0.66	gAvB	0.52
S2	gAvB	0.61	bAvB	0.45
S3	bAvB	0.63	gAvB	0.58
S4	gAvB	0.75	bAvB	0.50
	*Original (spatial)**		*Nearby objects*	
S1	bAvB	0.66	gBvC	0.50
S2	gAvB	0.61	bBvC	0.50
S3	bAvB	0.63	gBvC	0.48
S4	gAvB	0.75	bBvC	0.60
	*Original (spatial)**		*Relative to the door*	
S1	gCvD	0.68	bAvD	0.43
S2	bCvD	0.70	gAvD	0.55
S3	gCvD	0.61	bAvD	0.53
S4	bCvD	0.63	gAvD	0.38

g=green room; b=blue room; *all significantly above chance

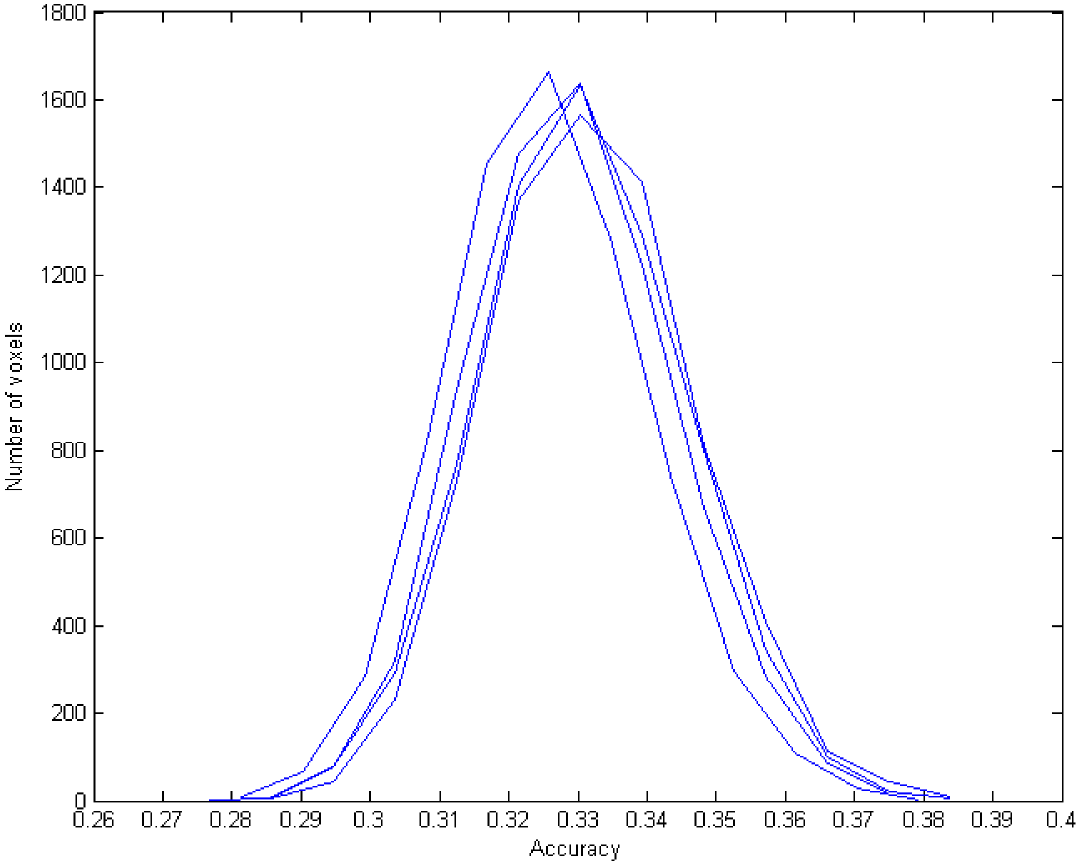

Figure S1. 95th Percentile Threshold for Each Voxel Individually

For each voxel in the search space (Figure 2B) the 95th percentile accuracy value across the 100 random runs for the 4-way classification in the blue room was taken and plotted as a population. Each blue line represents a single participant's population. The x-axis shows the 95th percentile accuracy value for a given voxel, and the y-axis displays how many voxels had 95th percentiles at that value. Populations were normally distributed for all participants (s1: mean 32.93%, SD 1.45%; s2: mean 33.20%, SD 1.48%; s3: mean 33.07%, SD 1.48%; s4: mean 32.59%, SD 1.46%) demonstrating there was very little variation in the signal-to-noise ratio across the voxels in the search space and therefore justifying our use of a single global threshold accuracy value for reprojection.
